# Implementing a scoping review to explore sport officials' mental health

**DOI:** 10.3389/fspor.2024.1436149

**Published:** 2024-07-29

**Authors:** Tori B. Carter, Paul Gorczynski, Christopher J. Coady, Ian J. Cunningham, Duncan R. D. Mascarenhas, Murray Grant, Philip Sullivan, Tom Webb, Lori A. Livingston, David J. Hancock

**Affiliations:** ^1^ School of Human Kinetics and Recreation, Memorial University of Newfoundland, St. John's, NL, Canada; ^2^ School of Human Sciences, University of Greenwich, London, United Kingdom; ^3^ School of Applied Sciences, Edinburgh Napier University, Edinburgh, United Kingdom; ^4^ School of Psychology, University of East Anglia, Norwich, United Kingdom; ^5^ Faculty of Applied Health Sciences, Brock University, St. Catharines, ON, Canada; ^6^ Faculty of Business and Law, Coventry University, Coventry, United Kingdom; ^7^ Faculty of Health Sciences, Ontario Tech University, Oshawa, ON, Canada

**Keywords:** referee, mental illness, well-being, sport policy, health

## Abstract

**Introduction:**

Sport officials are tasked with applying rules, maintaining fairness, and ensuring athlete safety. However, sport officials experience anxiety, burnout, and non-accidental violence, with the incidence of these events increasing worldwide. This has led to rising attrition rates among sport officials, with many sport organizations concerned for their operational capacity. The effects of anxiety, burnout, and non-accidental violence might contribute to or be indicative of sport officials' negative mental health outcomes. To develop a clear understanding of how sport officials' mental health is affected by their occupation, it is necessary to identify the mental health outcomes and predictors they experience, and to what extent. The purpose of this scoping review was to identify and examine the empirical research and policy documents surrounding sport officials' mental health.

**Method:**

One thousand, two hundred six articles were identified across four databases: PubMed, Web of Science, SportDiscus, and PsycINFO. Next, a policy search was conducted on the respective international governing body websites from English-speaking countries for the 60 included sports. Following screening, 18 studies and one policy document met the inclusion criteria for exploring sport officials' mental health.

**Results:**

Participants (*N* = 7,941) in the studies were mainly European male soccer and basketball referees. Most studies utilized quantitative inquiry (*n* = 15) rather than qualitative methods (*n* = 2) or framework development (*n* = 1). The research demonstrated that sport officials frequently experienced negative mental health outcomes and predictors including anxiety, depression, burnout, lower mental health literacy, and high levels of stigmatization towards mental health.

**Discussion:**

These outcomes were influenced by gender/sex, age, and experience. There is a need to explore personal and environmental (including occupational) factors that cause or contribute to sport officials' mental health symptoms and disorders.

## Introduction

According to the World Health Organization (1), optimal mental health is “a state of well-being in which the individual realizes his or her own abilities, can cope with the normal stresses of life, can work productively and fruitfully, and is able to make a contribution to his or her community” (p. 10). Mental health is a key aspect of human health and functioning that encompasses more than just mental disorders and illness, though this is a notable concern. Over the past two decades, mental health has become a key health concern in many sectors, including health care, business, and sport (2).

Historically, the sporting world has championed values of toughness and aggression and frowned on weakness, which contributes to a stigma associated with mental health in sport (3). Many sport organizations now promote and raise awareness surrounding athletes' mental health, yet these initiatives are not extended to sport officials (i.e., referees, judges, and umpires). Sport officials work in high-pressure environments where they are tasked with making critical decisions while ensuring athlete safety and fair competition. Regrettably, abusive behavior towards sport officials has been perpetrated by the media, spectators, athletes, coaches, and even the very organizations they represent (4). In fact, sport officials continue to face verbal abuse, harassment, and deliberate acts of violence; for instance, 92% of ice hockey officials in Canada had aggressive behaviors directed toward them (5), while 93% of soccer referees in England (6) and 68% of soccer referees in France were targets of abuse (7). Meanwhile, sport officials are often expected to accept maltreatment as part of their job. The cumulative effects of maltreatment and workplace pressures might have severe negative consequences on sport officials' mental health, which could influence intentions to discontinue.

Attrition is the rate of employee loss over time, and a low attrition rate is often an indicator of good organizational culture and a productive workforce (8). Over the past few decades, sport officials' attrition rates have left many youth and recreational sports leagues concerned about their operational ability. Research on Canadian sport officials' attrition rates shows there has been a 27% decline in the number of ice hockey officials in the past decade (9, 10). Similarly, the number of active soccer officials in Canada declined by 38% between 2016 and 2021 (11). On a broader scale, there are ongoing reports of annual attrition rates exceeding 20%–35% for various sports around the world (12). While several reasons for these sport officiating attrition rates exist, researchers have speculated that sport officials' deteriorating mental health is a key contributor (13). This is a common organizational concern in many occupations as mental health symptoms and disorders can influence absenteeism/presenteeism, reduce productivity, and increase voluntary turnover and long-term disability (14). Organized sports cannot exist without officials, and if organizations do not have the capacity to staff their competitions with qualified officials, it would reduce opportunities for athlete development and youth sport participation. As such, it is important to uncover how participating in this role affects sport officials' mental health. Doing so could lead to improvements in sport officials' working conditions and retention, while offering support to those who need it (13).

Researchers have investigated several concepts related to sport officials' mental health including anxiety (15, 16), depression (17), burnout (18–23), psychological well-being (24, 25), emotional intelligence (26, 27), mental health literacy (13, 28–30), and the influence of demographic factors (3, 26, 27, 30, 31). Further, research exists on the stigma surrounding sport officials' mental health, which might exacerbate the struggles officials experience while also rendering them reluctant to seek much-needed support (30). As such, providing evidence-based information to sport officials and organizations might help them to enhance their literacy of mental health issues and, in turn, develop solutions for the self-management of mental health.

Sport officials are often overlooked as a part of the sport system and their mental health is no exception. Given the current attrition rates in this profession, and the rising awareness of mental health issues in sport officials, it is timely to conduct a comprehensive review of current literature. Notably, we use the term “profession” here as the task of sport officiating is construed as a part- or full-time job; thus, organizations likely have the responsibility to ensure occupational health and safety for sport officials. By conducting a scoping review, we can collate research and policies that exist on this important topic. Therefore, the purpose of this study was to summarize the current knowledge of sport officials' mental health from an academic and organizational perspective. Two specific research questions included: (a) What is known about sport officials' mental health outcomes and predictive variables? and (b) What are organizations doing to support sport officials' mental health? A holistic search was essential to identify a broad spectrum of empirical and organizational information on sport officials' mental health. This review aimed to identify and describe the available studies and policies, highlight any gaps or limitations in the current body of evidence, and provide an overview of the state of knowledge in this specific area. In doing so, we endeavored to shed light on the existing knowledge pertaining to sport officials' mental health, potentially informing future studies and interventions aimed at addressing this critical aspect of their health and performance.

## Methods

This scoping review followed the five-step methodological framework proposed by Arksey and O'Malley (32). The steps included: (a) identifying the research questions, (b) identifying relevant studies, (c) study selection, (d) charting the data, and (e) collating, summarizing, and presenting the results. Given the limited number of studies in this field, we offer more depth in the results than a standard scoping review—akin to a systematic scoping review (33)—in order to present results that sport officials and organizations might find useful to improving their mental health.

### Identifying the research questions

Derived from the broader study research questions, the specific scoping review research questions were, “What empirical evidence exists regarding sport officials’ mental health outcomes or predictors?” and, “What organizational policies exist that target sport officials’ mental health?” These questions were created through collaborative discussion between nine sport partners (i.e., representatives from national and provincial organizations across Canada whose portfolios included overseeing officiating (e.g., Director of Officials) and academics (i.e., the authorship team) with expertise in both mental health and sport officiating.

### Identifying relevant studies

To begin, the research team (led by the first author) focused on identifying published research studies as a source of information and were guided by Gorczynski et al. (34) previous scoping review. The process included searching four databases in July 2023: PubMed, Web of Science, SPORTDiscus, and PsycINFO. Within each database, we searched the following terms: mental health, mental well-being/wellbeing, mentally ill, mental disorders, mental illness AND sport AND official, referee, judge, umpire (i.e., the first search was “mental health” AND “sport” AND “official”; the second search was “mental health” AND “sport” AND “referee”, etc.). Published studies were included if they were written in English and related to mental health outcomes or predictor variables among sport officials; this included articles, reviews, books, chapters, dissertations, theses, and abstracts. We did not impose limits on publication date. Any publication meeting the aforementioned criteria was added to a repository and the reference lists of all studies in the repository were searched—though no additional studies were identified for inclusion.

The second main search (led by the sixth author) targeted sport policy documents. Herein, we began by identifying all the international sport governing bodies listed on the Olympic website (*N* = 60). Next, we searched the respective websites of those international governing bodies, seeking any policy documents that related to sport officials' mental health. We repeated this process for all national governing bodies from each of the international organizations identified in the original search where English was their official language or one of their main languages and that send athletes to the Olympics.

### Study selection

**Figure 1:**
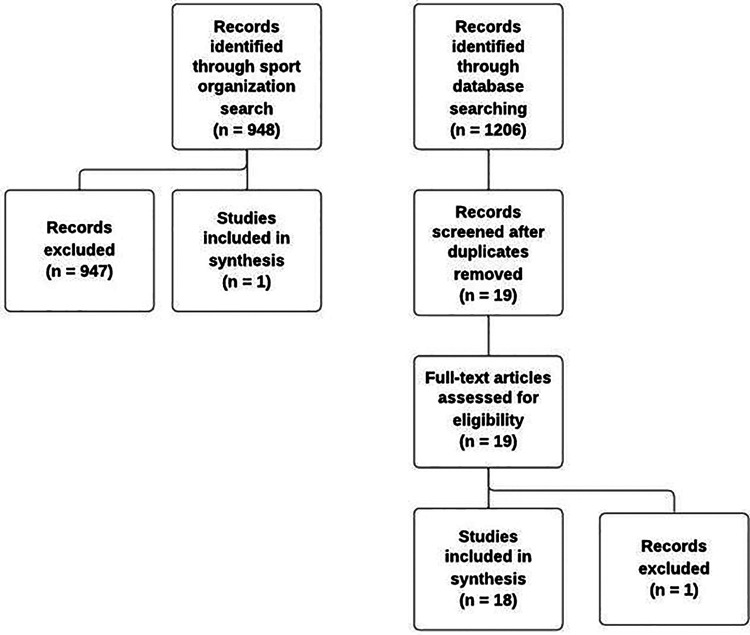
Records identified, screen, and included/excluded.

Initially, 1,206 academic articles were identified through database searching. The first author began by removing all duplicates. The remaining articles were subject to a title and abstract screening, with any studies that were clearly not related to sport officials’ mental health being removed. This left 19 articles that went through full-text screening. The first author and third author independently reviewed each article to ensure it met the inclusion criteria. When disagreements arose, the two authors consulted the senior (i.e., 10th) author to reach a consensus. Only one article was excluded, leaving 18 articles to be charted (Figure 1).

We followed a similar process to explore sport policy documents. The sixth author created a list of sport governing bodies from the 60 Olympic sports listed on the International Olympic Committee website (35) as of November 2022. Next, the sixth author compiled a list of countries where English is an official or main language and also those that send athletes to the Olympics. For every sport included in the list, the sixth author searched for relevant policy documents included on the respective international governing body website. For every country included in the list, the sixth author searched each national sport organization (i.e., for the 60 included sports) for relevant policy documents on their websites. Documents were relevant when the focus was on sport officials and the content included anxiety, worry, coping, abuse, harassment, non-accidental violence, mental disorders, mental health, mental illness, mental well-being, or burnout. When documents appeared to meet the inclusion criteria, the content was scanned further to make a final decision. Out of 948 documents that were identified, only one pertained to sport officials' mental health (Figure 1).

### Charting the data

For the published literature, the first and third authors used a data charting form to store extracted information on each identified article. Extracted information included author(s), year, journal, volume, sample size, sex, sexuality, race, nationality, experience, sport officiated, study location, and key findings pertinent to mental health. Since the search of policy documents yielded only one result, there was no formalized extraction process. The first author reviewed and compared extraction results for consistency and made modifications where necessary.

### Collating, summarizing, and presenting the results

Articles were categorized by methodology (i.e., qualitative, quantitative, mixed methods, review) and then data collection type (e.g., interview, survey, etc.). Demographic factors were summarized next, allowing us to detail the study sample size, age/experience, sex/gender, race/ethnicity, etc. Key attributes from each article were explored to document the main findings. Through this process, we identified several key results relevant to sport officials' mental health including burnout, anxiety, emotional intelligence, and mental health literacy.

## Results and discussion

Before outlining the main results, we summarized the 18 studies to provide a description of the type of research that has been conducted, along with the nature of the participants who were studied (Table 1; further expanded on in Appendix A). This section offers initial insights into the existing literature, with a broad discussion on the state of the literature to follow.

**Table 1:** Descriptive summary of the academic articles included within the scoping review.

Characteristic	Findings
Research method	Quantitative (*n* = 15); qualitative (*n* = 2), developing a framework for officiating (*n* = 1)
Data collection technique	Surveys/questionnaires (*n* = 15); interviews (*n* = 2)
Total participants	17 studies reported participant numbers *N* = 7,941; male = 7,356 (92.63%); female = 582 (7.33%); non-binary or other = 3 (0.04%)
Sexuality	One study reported on sexuality 286 heterosexual; 27 gay, lesbian, or bisexual
Disability	No studies reported on disability
Nationality	16 studies reported on participants’ nationality: Represented in two studies: United States, France, Norway, and Spain Represented in one study: Australian, Belgium, Brazil, Canada, Finland, Germany, Ireland, Italy, Jordan, Russia, Scotland, Sweden
Race/Ethnicity	No studies reported on race/ethnicity
Mean years’ experience	10 studies reported on officials’ experience 11.34 years on average
Age range	Eight studies reported on age range 14 to 77 years
Competitive level	17 studies reported competitive level Studies included participants who officiated professional (*n* = 4), college/university (*n* = 2), varsity high school (*n* = 2), and amateur (*n* = 1) sport; nine studies included participants from more than one competitive level
Sport	18 studies reported sport type Sports that appeared in multiple studies: soccer (*n* = 11), basketball (*n* = 6), handball (*n* = 2) Sports that appeared in one study: handball, Gaelic football, rounders, camogie, volleyball, rugby, field hockey, cricket, netball, Australian rules football, boxing, athletics, and lacrosse

### Burnout

Burnout is defined as a state of mental, emotional, and physical exhaustion that affects an individual's work capacity (18, 36). Six studies in total examined burnout. An investigation involving soccer, basketball, handball, and volleyball referees (*N* = 120) aimed to understand the relationship that experience, competitive level, and sport had on burnout (18). Over 70% of participants experienced moderate to high levels of burnout. Less experienced referees also had higher levels of burnout than more experienced referees. No significant relationships were detected for competitive level or sport. A second study investigated burnout among amateur and professional soccer referees [*N* = 36; (1)]. Referees working in amateur leagues developed higher rates of burnout than referees in professional leagues. Further, higher burnout rates led to higher attrition and negative career expectations.

Orviz-Martinez et al. (20) explored how verbal and physical aggression influenced burnout among grassroots soccer referees (*N* = 203). The authors found that the environment of grassroots soccer matches (e.g., conflict with players) and exposure to verbal or physical aggression contributed to referees' emotional exhaustion, cynicism, and decreased sense of effective refereeing. As shown by Sirin and Döşyılmaz (22), burnout can influence soccer referees' (*N* = 80) in several ways. Results showed that marital status, referee role (i.e., head vs. assistant), and refereeing experience all had a significant relationship to burnout. Married referees were found to have higher levels of intrinsic satisfaction and were less susceptible to burnout. Similar trends were seen for assistant referees when compared to head referees. Lastly, more experienced referees were less susceptible to burnout in comparison to less experienced referees. No relationship was found between age, occupation, educational status, and burnout. A significant negative association was found between job satisfaction and burnout—when burnout was higher, job satisfaction was lower.

A study on basketball referees (*N* = 721) examined the relationship between sources of stress, burnout, and intentions to quit (21). Results revealed five main sources of stress: (a) performance concerns, (b) fear of physical harm, (c) lack of recognition, (d) time pressure, and (e) interpersonal conflict. These factors, aside from fear of physical harm, were mildly related to referee stress. On occasion, performance concerns, interpersonal conflict, and time pressure contributed to burnout experiences. These factors were also deemed to moderately contribute to referees' intentions to quit.

Several other factors contributing to burnout (e.g., role-culture conflict, fear of failure, time pressure, interpersonal conflict, fitness concerns) were identified in a study on soccer referees [*N* = 529; (23)]. Results showed a direct positive relationship between stress and burnout. The inability to assert control as an official and role-culture conflict (the mismatch between the expected and perceived appreciation and recognition in the environment) contributed to increased burnout. Burnout in soccer referees also appeared to have indirect effects on turnover intentions. Further, fear of failure, role-culture conflict, and interpersonal conflict had indirect effects, through burnout, on turnover intentions.

It was clear from the literature that burnout affected many sport officials. Notably, exposure to abuse and role-culture conflict (e.g., expecting to be treated well, yet being abused) led to increased perceptions of burnout. This finding is not surprising, as being maltreated in one's profession likely leads to mental, emotional, and physical exhaustion. Further, less experienced, grassroots, and unmarried sport officials reported more burnout than experienced, professional, and married officials. Perhaps sport officials learn effective coping skills as they gain experience and move up through the ranks, while also developing stronger support networks (e.g., a spouse) to insulate themselves from abuse. Similar findings exist in studies examining burnout in other professions, where burnout was twice as likely in hospitals with inexperienced nurses than those with experienced staff (37).

### Psychological well-being

Four studies demonstrated how sport officials' psychological well-being (i.e., constructs such as life satisfaction, feelings of accomplishment, self-acceptance, and sense of purpose) seems to be affected by their occupation. This was demonstrated in a study on officials from various sports [*N *= 317; (29)]. The inability to self-manage workload and demonstrate professional autonomy were strongly associated with negative emotional symptoms and psychological well-being. Demographic factors also influenced psychological well-being; sport officials who were younger, were not in a committed relationship, had lower levels of education, and had less officiating experience reported higher rates of negative emotional symptoms. Meanwhile, sport officials who were male, were older than 50 years, were in a committed relationship, and had more officiating experience reported higher rates of psychological well-being (29). A similar conclusion was drawn from a study of 438 officials from across several sports (28) where experiencing physical abuse was associated with lower levels of psychological well-being. Subsequently, higher distress was associated with poorer mental health and greater intentions to quit.

Kim et al. (25) examined basketball referees' (*N* = 410) authenticity at work (being one's true self at work) and engagement (as predicted by seven factors: administrator consideration, intrinsic motives, mentoring, remuneration, sense of community, lack of stress, and continuing education) as antecedents for psychological well-being. Experiencing negative social interactions (e.g., verbal abuse) with other sport actors (e.g., coaches, athletes, fans), had a negative influence on referees' engagement; specifically, referees who had more negative interactions were more likely to quit. Further, authenticity in interactions with other sport actors positively influenced engagement. Thus, the positive relationship between authenticity and engagement might relate to psychological well-being.

Another study followed soccer referees (*N *= 391) over the course of one season (24). Referees who reported lower social support were more likely to present symptoms of common mental disorders. Referees who lacked social support also reported symptoms of disordered eating, indicating there might be a relationship between social support and eating disorder prevalence. Nearly half of the participants reported symptoms of common mental disorders at baseline. Further, those referees who were suffering from injuries were three times more likely to report symptoms of anxiety and depression.

Sport officials' psychological well-being was negatively affected by several factors: inability to manage workload, lack of autonomy, low social support, and abuse. Resoundingly, these factors identify the need for organizations to offer more support to their officials. Such supports might be especially important for young, inexperienced, and single (i.e., those without a committed partner) sport officials, who demonstrated lower psychological well-being. In fact, social support (including organizational support) is a critical factor in mental health, and researchers have consistently demonstrated the importance of social support in maintaining good mental health (38).

### Anxiety

Given the high-pressure work environment in which sport officials operate, anxiety is a frequent outcome. Two studies examined anxiety. Johansen and Haugen (15) studied this in 83 elite soccer referees. Top-tier referees experienced significantly higher anxiety than second-tier officials. Anxiety was also negatively related to self-confidence; that is, referees who reported low self-confidence (most frequently being unsure about the accuracy of their decisions) also reported higher anxiety. The sporting environment can act as an additional stressor in triggering sport officials' anxiety. This was investigated in a sample of 30 basketball referees (16). Results identified that referees' reported anxiety could be influenced by external factors like crowd noise. Specifically, referees with high competitive anxiety exhibited poorer performance in pressing crowd conditions.

A key finding was that higher-level sport officials experienced significantly higher anxiety than lower-level officials. Often, these sport officials are put under more pressure within their environments—particularly with respect to making accurate decisions in front of large audiences—which might act as an anxiety trigger. This is amplified when sport officials' are performing with low confidence. Further, sport officials' anxiety is influenced by crowd noise (16). Thus, it appears that external sources play an integral role in sport officials' anxiety.

### Emotional intelligence

Emotional intelligence (EI), refers to an individual's ability to perceive, express, understand, and manage their emotions (39). This was researched in two studies found in the scoping review. To explore EI among sport officials, Louvet and Campo (27) recruited 139 male soccer referees and found that, compared to non-elites, elite soccer referees reported higher rates of EI. Further, multiple regression analysis revealed that higher scores on EI were indicative of positive task-oriented coping—this relationship was particularly strong in the facets of emotionality, sociability, and self-control (27). EI has also been described as a factor that can help protect against negative mental health outcomes. Specifically, Fernández et al. (26) investigated EI as a factor influencing the mental health of sport officials (*N* = 4,099) from several sports. According to the results, there was a clear relationship between EI, burnout, and mental health; referees with higher EI scores were less affected by the negative consequences associated with burnout and had a lower frequency of mental health symptoms (26).

EI has been shown to be a protective factor against negative mental health outcomes (40), which was the case for sport officials as well. Finding that elite sport officials demonstrated higher EI offers insights. Specifically, it might mean that, through their experiences, elite sport officials develop the necessary EI (e.g., positive task-oriented coping) to maintain good mental health. Therefore, providing sport officials with tools to develop EI might insulate them from burnout and negative mental health symptoms. Encouragingly, previous trials have shown EI training to be effective in enhancing several dimensions of EI including emotional understanding and management (40).

### Mental health literacy

Mental health literacy is the knowledge and beliefs people have about mental disorders that can help recognize, manage, and prevent them (41). Gorczynski and Thelwell (30) conducted the sole study in this area, focused on soccer referees' (*N* = 313) mental health literacy. Soccer referees reported low rates of mental health literacy; in fact, when compared to previous research on athletic staff (42)—which used the same scales—sport officials reported lower rates of mental health literacy than athletes (30). However, when comparing participants' sex, the authors found that female sport officials had significantly higher mental health literacy than males. The study also revealed many negative attitudes expressed towards others living with poor mental health, which the authors described as mental health stigma.

Low mental health literacy among sport officials might be attributed to the stigma surrounding mental health—especially within males. Research has shown that the stigma among males surrounding mental illness poses a barrier to help-seeking and the use of services, and also diminishes social connection between peers (43). Further, sport organizations depreciate mental health issues as unwelcomed weaknesses, further contributing to barriers in help-seeking behaviors (44). This likely fosters an environment where a sport official feels it is unacceptable to seek help regarding their mental health. If this is the case, a young sport official might not feel comfortable inquiring about how to build resiliency to negative interactions with coaches and athletes. This is a troubling trend that should occupy the thoughts of sport organizations.

### Female sport officials' mental health

Two studies have investigated the mental health of female sport officials. First, 20 female basketball referees were interviewed about gendered aggressions and mental health (3). Eighteen of the 20 referees indicated they had experienced gender-related macro- and micro-aggressions, which negatively impacted their mental health (increased anxiety and fear; decreased self-esteem). Furthermore, 12 participants specifically mentioned the stress that existed because the sport officiating structure often operated like an “old boys club”. Moreover, participants recounted instances where they had been sexually objectified in the role and felt they were held to unrealistic expectations of how they should look, which sometimes led to body image issues. Such negative body image perceptions were linked to poor emotional regulation skills, which might affect EI (3).

Similar trends were found in a study that examined female English soccer referees' mental health (31). Semi-structured interviews of 12 referees revealed toxic, abusive, male-dominated environments that included sexist and derogatory language, negatively affecting participants' mental health. The notion of an “old boys club” was prevalent in several interviews with participants believing that female referees were seen as less physically capable of progressing to high levels of sport officiating. This aligned with a second theme, where derogatory language and sexist comments were frequently directed at female officials, which negatively affected their mental health. Further, feelings of isolation were reported due to a lack of formal and informal support networks between referees and their organizations. Finally, all of these experiences were exacerbated because referees received fragmented information and training, despite progressive societal movements.

Officiating, like many other aspects of sport, is a male-dominated profession. The literature reviewed aligns with this as only 7.33% of participants were female. Within the studies, female sport officials reported overarching themes of sexism, discrimination, negative body image, and isolation, which contributed to negative mental health outcomes. Sadly, female sport officials face similar challenges as female athletes [e.g., sexism and discrimination; (45)], indicative of a sporting environment needing to change. Recent initiatives have shown that multiple factors contribute to female sport officials' intentions to quit [e.g., abuse, sexism, and lack of support; (46)]. Such initiatives could be leveraged to begin addressing and improving female sport officials' mental health.

### Policy document

The lone policy document on sport officials' mental health was published by the Gloucestershire Football Association (47). The webpage described the football association's (FA) guidance toolkit, which was comprised of three documents: (a) FA Mental Health Guidance Notes for Referees (48), (b) FA Mental Health Guidance Notes for Referees Looking After Yourself (49), (c) FA Mental Health Guidance Notes for Referees Mental Health Conversation Tips (50). The first document is 32 pages long, and outlined the definition of mental health, how referees and those who support them can spot the warning signs of negative mental health, and how to support each other to get help (48). The second document provided referees with ways to look after themselves, and a referee testimony on dealing with mental health concerns (49). The final document focused on how to talk about mental health (starting, maintaining, and closing the conversation) and provided the reader with language tips to keep in mind when talking about mental health (50).

The lack of policy surrounding sport officials' mental health is indicative of the avoidance of organizational responsibility. The evidence from this review clearly outlines a severe need for organizational action. There is a total lack of any organizational responsibility to keep their workers (i.e., sport officials) safe—physically and mentally—from abuse, harassment, bullying, and violence. Likely, in most countries, organizations have a legal responsibility to ensure this and steps ought to be taken immediately to rectify the situation.

## General discussion

The purpose of this review was to summarize the current understanding of sport officials' mental health outcomes and predictive variables. This included investigation into both academic research and sport organization policies to form a complete picture on what we know about sport officials' mental health, and what, if anything, is being done to ensure their occupational safety concerning mental health. This section will unpack the nature of the research designs, the nature of the results, actions for sport scientists and organizations, and limitations of the current study.

### Nature of the research designs

A key element of a scoping review is to document and comment on the various research designs used in previous studies, in an effort to highlight methodological gaps and provide direction for future studies. With only 18 studies, research on sport officials' mental health concepts is very limited. Further, the methods used to investigate the topic are limited. Only two studies availed of qualitative designs (interviews), while 15 studies utilized quantitative designs (all surveys). Each of these is considered a limitation of the research field. First, the lack of qualitative designs indicates an incomplete view on sport officials' personal stories, reflections, and experiences of negative mental health outcomes, which are imperative to truly understanding this issue. Second, the reliance on surveys overlooks other quantitative methods (e.g., observational analysis and interventions) that provide deeper insights into sport officials' mental health. Researchers ought to consider such methods with future explorations.

Whereas researchers of sport officials’ mental health outcomes/predictors should be commended for the breadth of topics studied, including burnout, psychological well-being, anxiety, EI, and mental health literacy, a closer look at the literature reveals several gaps and shortcomings regarding the demographic variables of participants in these studies. First, participants were 93% male—a common limitation in sport officiating research (51). Assuming that male and female sport officials have the same experiences with mental health is ill-advised, as supported by the findings herein (3, 31); therefore, researchers need to pay particular attention to female participants to complement our existing understanding of officials' mental health. Second, most studies examined soccer and basketball officials. Given the limited literature on various sport official populations, we cannot dissertate that these experiences are shared in similar severity and frequency among officials in other sports. Third, most studies were conducted in Europe; it is unclear if cultural factors in other regions impede or improve sport officials' mental health. Finally, related to cultural factors, none of the articles exclusively explored minority sport officials' (e.g., race, ethnicity, sexuality, and disability) mental health experiences. Understanding these and other sociocultural demographics may assist in promoting a diverse and equitable sporting environment and ensure all individuals in the profession are protected.

### Nature of the results

From the scoping review, it is evident that, to some extent, sport officials have experiences that predict or lead to negative mental health outcomes (e.g., burnout, low psychological well-being, and anxiety). In addition, sport officials report varying EI and low mental health literacy. Certainly it is plausible that these findings are attributed to sport officials' workplace stressors (e.g., task demands and time commitments) and negative interactions with other sport actors (i.e., athletes, coaches, and spectators), which could lead one to conclude that officiating has a negative impact on one's mental health. None of the reviewed studies, however, can unequivocally support this conclusion. It might be that sport officiating exacerbates officials' existing negative mental health outcomes, or more simply, officials experience negative mental health outcomes just as one would expect from individuals in other professions. What is clear, though, is that sport officials' mental health is a serious occupational concern and organizations should introspect on whether their policies and practices support officials' mental health. Further, researchers ought to continue investigations in this area to ascertain the extent to which mental health is affected by working as a sport official.

The results and discussion section offers insights into the individual findings of the articles, grouped by studied variables. What is lost in this approach, however, is how the variables might interact. A few of the reviewed studies examined multiple variables [e.g., EI and burnout; (26)], but here we offer insights into how these variables might interact more broadly. Considering the collective results, it is likely that certain stressors (e.g., abuse and crowd noise) can trigger negative mental health outcomes in some sport officials. For instance, if a sport official experiences elevated and persistent anxiety, it stands to reason that psychological well-being—and potentially burnout—are also negatively affected. The same is true if one experiences sustained decreases in psychological well-being—anxiety and perceptions of burnout might then increase. As such, negative mental health variables likely influence other variables, which could magnify overall outcomes. Whereas all sport officials likely encounter stressors at some point in their careers, only some experience negative mental health. Therefore, it seems that the extent to which such outcomes are experienced depends on protective factors possessed by sport officials—specifically, EI and mental health literacy. Furthermore, younger age, less experience, and smaller social support networks appear to be risk factors for negative mental health among sport officials. This all demonstrates how related and interconnected the studied variables are with respect to sport officials’ mental health outcomes and predictors. Clearly, sport organizations need to ensure training and support for their officials, especially those who might be more prone to negative mental health.

### Actions for sport scientists and organizations

In conducting this scoping review, we have identified several areas for future research that sport scientists can consider, all of which would enhance our understanding of sport officials' mental health. Specific actions include:

• Complement existing literature with new research that specifically targets sport officials who are underrepresented in the mental health literature. This includes females, minority groups (based on sexuality, race, ethnicity, and disability, for instance), officials in sports other than soccer and basketball, and those outside of Europe.
• Allow study methods to be dictated by pertinent research questions, rather than relying on surveys to collect data. This might broaden research to include focus groups, observational analysis, and interventions.
• Further examine the relationships between sport officials' burnout, psychological well-being, and anxiety to understand how these variables influence each other. Linking these constructs to sport officials' attrition would be beneficial.
• Study variables (e.g., EI, mental health literacy, and coping strategies) that might insulate sport officials from negative mental health outcomes. More insights on such variables could lead to evidence-based educational opportunities that support mental health.
• Consider investigating specific mental illnesses. The missing knowledge concerning the clinical aspect of mental health presents an opportunity to further our understanding of any potential discrepancies in the prevalence of mental disorders between the general and sport officiating populations. Utilizing validated screening questionnaires and psychological assessments from a health professional (psychologist or psychiatrist) would be useful in estimating the prevalence of these disorders among sport officials. This research would be essential in furthering the initiative of promoting sport officials' mental health.
• Utilize educational programs from bullying and cyberbullying in schools and apply it to the sporting world. Previous programs have focused on the early detection of cyberbullying on social media, and teaching individuals (victims, bullies, and bystanders) on how to cope with cyberbullying and improve resilience (52). This issue extends beyond the world of sport scientists; occupational epidemiologists should take action here in researching this issue further through an occupational health lens. Interdisciplinary collaboration on research efforts surrounding the mental health of sport officials from psychologists, epidemiologists, and sport scientists would optimize the ability of positive outcomes for this group.

Similarly, this scoping review is well-suited to developing recommendations for sport organizations, with the goal of supporting sport officials' mental health. We argue that, as employers or supervisors of sport officials, it is incumbent upon organizations to take the lead in implementing support mechanisms. Specific actions include:

• Offering ongoing professional development opportunities that teach sport officials about various aspects of mental health (e.g., burnout, anxiety, and psychological well-being). These educational opportunities should be designed to support sport officials in developing tangible strategies that reduce negative mental health outcomes, and could include face-to-face or online platforms.
• Facilitate social support networks within sport officials, especially within marginalized groups like women who have a higher predisposition to social support (38), which could facilitate positive mental health outcomes in the population. In such support networks, organizations should ensure an organizational representative and a diverse group of sport officials who are appointed by the administration.
• Ensure that sport officials in these high-pressure environments are provided sufficient organizational support before, during, and after competitions. This support should include having a designated support person for sport officials to contact and available resources that go beyond professional development opportunities.
• Take initiative in reducing the stigma surrounding mental health in sports. Broad messaging targeting all sport participants is essential in ensuring the message is clear; mental health is a priority, not a weakness. This may be effectively done throughout the development of aforementioned support systems.
• Create policies that ensure marginalized sport officials are supported and feel a sense of belonging in their roles. This includes developing policies that reduce abuse and discrimination, creating social support networks, and conversing with marginalized sport officials to understand their experiences.
• Develop preventive policies aimed at eliminating bullying, harassment, abuse, and violence directed towards sport officials. Outside of organizational control lies the abuse sport officials face through online forums and social media. This, in all likelihood, has an impact on mental health and well-being, just as face-to-face abuse/aggression does. While an organizational policy cannot directly eliminate this cyber-bullying, if the organizations are to take the first step in stopping the normalization of abuse towards sport officials, it might lead to change from spectators, media, and other sport participants.

### Limitations of the current study

Two main limitations are noted for this scoping review. First, as is typical with scoping reviews, we did not exclude studies based on research quality. As such, studies of high and low quality equally contribute to the results presented herein, and the subsequent conclusions. Second, our search was limited to English-only studies (due to the authors’ primary language). Therefore, studies published in other languages could offer insights into sport officials’ mental health outcomes and predictors, but are absent in this review. Nevertheless, in following a rigorous process, we believe this scoping review offers valuable insights that can direct future research and sport organizations.

## Conclusion

Through conducting a comprehensive search of relevant studies and policy documents, we have collated and summarized the current literature on sport officials' mental health. From this review, it is clear that sport officials are suffering negative mental health outcomes. Our investigation revealed a complex array of mental health challenges faced by sport officials, including burnout, anxiety, and psychological well-being. These challenges more strongly affected less experienced and grassroots sport officials, highlighting the need for targeted inventions and supports at this level. Additionally, the stigma surrounding mental health within the broader sport officiating community was identified as a barrier to help-seeking behaviors and positive mental health outcomes. Furthermore, female sport officials experienced marginalization, discrimination, and were at a greater risk of negative mental health outcomes. As it stands, it is expected that sport officials self-organize and self-manage their own support systems; from looking at the trends in the literature, this is clearly not working. A mentally healthy workplace is a safe workplace; beyond that, it creates a high-functioning, respectful, and productive environment. It seems that many sport officials lack a safe work environment. Without formalized support, it is not possible for organizations to expect the mental health outcomes of their employees (i.e., sport officials) to improve. This is an occupational health issue and must be treated as such.

The implications of these findings are far-reaching and underscore an urgent need for action at both organizational and research levels. Organizations, researchers, and sport participants must work to change the narrative around officiating culture. Organizational policies—including zero tolerance policies for abuse (53)—must be developed and implemented to address issues including bullying, harassment, and abuse, as well as to promote positive mental health practices and supportive environments. Abuse towards athletes has long been discouraged through the use of new technologies online, and this can easily be applied in the case of officials—as demonstrated by World Rugby through their use of artificial intelligence screening (54). Since abuse and other negative experiences contribute to sport officials' intention to quit (46), it is the responsibility of organizations to spearhead these interventions. Moreover, efforts to combat stigma and promote mental health literacy within the sport officiating community are essential for creating a safer, more accepting culture surrounding mental health and for protecting the capacity of organizations to recruit and retain officials.

Looking ahead, future researchers should prioritize interdisciplinary approaches and ensure a broader population is included within its studies; this includes factors such as race, ethnicity, sexuality, and gender identity to further understand how demographics influence sport officials' mental health experiences. Also, future studies should take on more applied designs that introduce and implement different intervention approaches to evaluate efficacy and fit to the needs of different sport officiating populations. This would include evaluating the role of media and cyberbullying on sport officials’ mental health and how organizations can lead the way in promoting positive mental health and literacy. Stakeholders in the sporting community can work to develop evidence-based interventions, enhance availability of trained mentors in mental health support, and create policies that promote inclusivity and challenge the traditional sporting norm that sport officials are disposable independent contractors. It is imperative that a shift in sporting culture be made to promote positive mental health among officials.

## Data Availability

The raw data supporting the conclusions of this article will be made available by the authors, without undue reservation.

## Appendix A

Summary of the Literature.

**Table:**

Author(s)	Sample	Sport(s)	Theme	Key Findings
Al-Haliq et al. (18)	120M	Soccer, Basketball, Handball, Volleyball	Burnout	• Referees had moderate levels of burnout. • Significant relationship between burnout levels and referees’ experience. • Less experienced referees had higher levels of burnout than did more experienced ones. • No significant relationships were detected between the levels of burnout of referees and the refereeing level and type of sport they refereed.
Brick et al. (28)	438 434M 4F	Gaelic Football, Rounders, Camogie, Handball	Psychological Well-being	• For verbal abuse, only the direct and indirect effects model achieved acceptable fit and significantly explained variance in mental well-being (9.4%), anxiety (15.2%), depression (15.6%), and intentions to quit (19.1%). • For physical abuse, though higher distress was associated with poorer MH and greater intentions to quit, none of the models fully explained the relationships between all variables. • Findings demonstrated a relationship between abuse, subsequent distress, and MH outcomes.
Carson et al. (29)	317 244M 73F	Rugby, Soccer, Field Hockey, Cricket, Netball, Australian Rules Football, Basketball, Boxing, Athletics, Lacrosse	Psychological Well-being	• Officials who were younger, not in a committed relationship, having lower levels of education, and less officiating experience reported higher levels of negative emotional symptoms, • Males, older than 50 years, in a committed relationship, and more officiating experience had higher levels of psychological well-being. • The ability to self-manage workload and demonstrate professional autonomy were strongly associated with negative emotional symptoms and psychological well-being. • Officials reported high negative emotional symptoms, but also high levels of psychological well-being.
Da Gama et al. (19)	36 32M 4F	Soccer	Burnout	• Referees working in amateur leagues may develop higher rates of burnout syndrome than referees in professional leagues. • These referees also showed faster problem solving. • Positive expectations were reduced over career time with amateur referees, and self-judgment appeared to increase.
Fernández et al. (26)	4,099 3,773M 326F	Soccer	Emotional Intelligence	• Emotional intelligence is a protective and moderating factor that can help to protect against burnout and health problems in referees.
Gorczynski and Thelwell (30)	313 268M 45F	Soccer	Gender	• The level of mental health literacy scores was lower than in previous studies. • There is a stigma associated with seeking help in this profession.
Gorczynski and Webb (13)	N/A	Soccer	Framework	• Limited research on sport official mental health has resulted in a deficit of knowledge of mental health symptoms and disorders in this population and also limits the ability to create evidence-based interventions.
Johansen and Haugen (15)	83 73M 10F	Soccer	Anxiety	• Results showed that referees in the premier league (high level) scored higher on anxiety compared to refs working at a lower level. • Low self-efficacy and self-confidence correlated to high anxiety scores. • Aggressive behaviour from players and coaches was an influencer in referee confidence, especially when it came to making proper calls. • Sports officials experience no more than a moderate amount of stress while working.
Kilic et al. (24)	391	Soccer	Psychological Well-being	• Those suffering from severe injuries were 3× more likely to report symptoms of anxiety and depression. • Low satisfaction of social support was closely related to reporting symptoms of eating disorders. • Nearly half the participants reported symptoms of CMD at the baseline.
Kim et al. (25)	410 385M 22F	Basketball	Psychological Well-being	• Referee engagement was not influenced by lack of stress rather that experiencing problematic social interactions with other sport actors were cited as reasons for attrition. • Referee authenticity at work positively influenced referee engagement. • Referee engagement was an antecedent for psychological well being.
Louvet and Campo (27)	139M	Soccer	Emotional Intelligence	• Higher level referees possess higher levels of emotional intelligence.
Orviz-Martínez et al. (20)	203 194M 9F	Soccer	Burnout	• The environment surrounding grassroots football matches and the level of verbal and physical aggression to which referees are exposed increase the factors of emotional exhaustion and cynicism, and diminish the sense of effectiveness of their refereeing.
Rainey (21)	721 664M 57F	Basketball	Burnout	• 5 main correlated sources of stress factors; performance concerns, fear of physical harm, lack of recognition, time pressure, and interpersonal conflict. • All factors besides fear of physical harm were seen as mildly related to their stress. • Performance concerns, interpersonal conflict, and time pressure, all consistently contributed to burnout experiences. • These moderately contribute to referees’ intentions to terminate refereeing.
Sirin and Döşyılmaz (22)	80M	Soccer	Burnout	• Referees were found to have high levels of job satisfaction. • Significant differences were found in job satisfaction levels and things like marital status, football experience and referee post. • There was no significant relationship between job satisfaction and educational status, occupation, duration of refereeing and age. • A negative and significant association was found between job satisfaction dimensions and burnout.
Sors et al. (16)	30M	Basketball	Anxiety	• Decisions of referees with high anxiety might be more easily influenced by external factors like crowd noise.
Taylor et al. (23)	529M	Soccer	Burnout	• Fear of failure identified related most strongly to feelings of burnout. • Age was negatively related to burnout. • Total perceived stress and burnout had only indirect effects on turnover intentions. • Stress had a direct negative effect on burnout while burnout appeared to have a direct positive effect on perceived stress over time.
Tingle et al. (3)	20F	Basketball	Gender	• Gendered aggressions negatively impact female referees. • Mental health issues are stigmatized amongst sport officials.
Webb et al. (31)	12F	Soccer	Gender	• Toxic, abusive, male-dominated environments that included sexist and derogatory language, negatively affect female sport official mental health. • Limited support for mental health. • Officials often experienced poor mental health during and after matches.
